# Water and Beverages Intake Among Workers Amid the COVID-19 Pandemic in Indonesia

**DOI:** 10.3389/fnut.2022.832641

**Published:** 2022-03-14

**Authors:** Diana Sunardi, Dian Novita Chandra, Bernie Endyarni Medise, Nurul Ratna Mutu Manikam, Dewi Friska, Wiji Lestari, Putri Novia Choiri Insani

**Affiliations:** ^1^Indonesian Hydration Working Group, Faculty of Medicine, Universitas Indonesia, Jakarta, Indonesia; ^2^Department of Nutrition, Faculty of Medicine, Universitas Indonesia – Dr. Cipto Mangunkusumo General Hospital, Jakarta, Indonesia; ^3^Department of Child Health, Faculty of Medicine, Universitas Indonesia – Dr. Cipto Mangunkusumo General Hospital, Jakarta, Indonesia; ^4^Occupational Medicine, Department of Community Medicine, Faculty of Medicine, Universitas Indonesia, Jakarta, Indonesia

**Keywords:** fluid intake, hydration, health workers, general workers, personal protective equipment

## Abstract

As the COVID-19 pandemic hit worldwide in the early 2020, people were urged to alter their behavior to prevent disease spread, thus, led to change water intake. This study aimed to analyze water and beverage intake among health workers and general workers during COVID-19 pandemic in Indonesia. This study was a comparative descriptive study where the participants were 20–45-year-old health workers and general workers in Indonesia. Data collected included demographic, water and beverage intake, physical activity, nutrient intake, and body weight and height. All data collection was obtained online using self-reported questionnaire. Water intake data was collected for 7 days consecutively using a 7-day fluid record. There were 246 participants comprised of 102 (41.5%) general workers and 144 (58.5%) health workers who were analyzed in this study. All participants showed barely adequate intake of daily total fluid 1,882 (1,473–2,433) ml/day. Total fluid intake among general workers was 1,759 (1,447–2,396) ml/day, whereas in health workers it was slightly higher 1,939 (1,516–2,446) ml/day (*p*-value = 0.378). Among health workers who were highly exposed to patients with COVID-19 showed the highest percentage in drinking water adequately (20 participants, 60.6%) compared to health workers who worked under moderate (29 participants, 48.3%) and low (24 participants, 47.2%) level of exposure to patients with COVID-19. In conclusion, workers need to improve their daily fluid intake. This study also showed better behavior of water consumption among health workers compared to general workers despite of their strict personal protection equipment during working hour.

## Introduction

Approximately 60–70% of adult human body composition comprises water. With the prominent function of water in the body, its presence is irreplaceable. Water acts as body fluid that forms cells, body thermal regulator, solvent in most reaction, lubricant, and transporter (1). Insufficient amount of water in body, even only for 2%, might develop many unfavorable conditions, such as the decline of psychomotor, visual motor, aerobic, and cognitive performance along with worsen alertness, fatigue, and mood (2–4).

In the long run, behavior of low water intake showed impacts in metabolism impairment. Low water intake was proven associated with kidney stone and urinary tract infection (5). Regular adequate water intake also acts as protective factor of chronic kidney disease (6). Furthermore, some studies showed association between adequate water intake and body weight improvement and lower risk of overweight (7–9). Enhörning et al. reported that water supplementation is potential for diabetes mellitus prevention among low drinker (10).

Since March 2020, WHO declared COVID-19 as global pandemic, and Indonesia declared the pandemic 6 days later as national disaster for growing number of COVID-19 cases among Indonesians (11, 12). The regulations for preventing COVID-19 transmission were released by government as a response of emergency condition that was declared by Indonesian National Board for Disaster Management (13). Students were obliged to study from home, and some workers in essential and non-essential sectors were required to work from home (WFH) (13). These new manners led people to show the alteration of behavior, especially for dietary and drinking habit (14).

The working pattern that requires flexibility of working time, duration, and location was not something new for few workers, yet most workers need adjustments of this method. The most notable alteration was less time for mobility. Furthermore, WFH increases the opportunity to experience longer time of leisure activity and sleeping. In the term of dietary behavior, people who WFH were more likely to spent more time to eat, drink, and undertake food production activity (15).

Other than contrasting working behavior, people were obliged to wear masks outside the house. For people who work from the office, this condition discourages them from drinking water because they have to take off their mask before drinking. This situation might be worse for health workers who were exposed to patients with COVID-19 and required to wear hazmat suits and other personal protective equipment (PPE) for around 6 h. Some changes in the working environment promote people to modify their drinking behavior as well. The aim of this study was to observe the water and beverage intake among the workers in Indonesia amid the pandemic.

## Materials and Methods

### Designs and Settings

This comparative descriptive study was conducted among healthcare workers and general workers in Indonesia. Data were collected by self-report questionnaire in April 1–15, 2021. We conducted recruitment and data collection by online process without meeting between participants and interviewers. Google Form was used as a tool for data collection where participants could input the data.

The recruitment was started at the end of April 2021 and distributed among workers in Indonesia over social media. Workers who completed the early questionnaire on the recruitment and met the eligibility criteria were contacted for further data collection. Early questionnaires were included demographic data, drinking behavior during pandemic, working environment, record of body weight and height, and hydration knowledge, attitude, and practice. By the mid of April, data collection was finished.

### Participants

We enrolled 305 participants and contacted 298 participants who were eligible; 246 subjects completed data collection of water and beverage intake. Eligibility criteria were workers resided in Indonesia, 20–45 years old, able to communicate online, and not having metabolic diseases related to hydration. Both general workers and health workers were expected to participate in this study. The minimum sample size was determined using a sample survey formula where insufficient drinking among adults referred to Laksmi et al. (16). Laksmi et al. as our reference investigating water intake among Indonesia population using a 7-day fluid record as we also used in this study (16). The formula used was shown below. We applied 10% margin of error, 95% confidence level, and considered possibility 20% of drop outs; thus, minimum participants needed for this study in each group was 91 participants. It resulted a minimum of 182 participants in total.


$$n=\frac{{\left(Z_{1-\alpha /2}\right)}^{2}\,P\,\left(1-P\right)\,N}{d^{2}\,\left(N-1\right)+{\left(Z_{1-\alpha /2}\right)}^{2}\,P\,\left(1-P\right)}$$

where α is the confidence level (95%), *Z*_*1–α /2*_ is the *Z*-score of confidence level (1.96), *P* is the incidence of inadequate water intake among adult in Indonesia (72%), *N* is the total population of health workers and general workers in Indonesia, *d* is the margin of error (10%), and *n* is the minimum sample required.

### Procedures

This research was approved by FKUI-RSCM (Faculty of Medicine Universitas Indonesia – Dr. Cipto Mangunkusumo General Hospital) Research Ethical Committee with protocol number 21-02-0214 and managed according to the Helsinki Declaration. Prior to data collection in the recruitment process, participants were required to read the research procedure and tick the “I agree to participate” of the informed consent section to continue to the data collection section. During recruitment and initial data collection, we collected the name, phone number, and their email address to contact the eligible participants to inform further regarding data collection of 7-day fluid record, food intake, physical activity, and urine color.

After being contacted by the research’s team, participants were given guidance on how to fill the form of water and beverage intake for 7 days consecutively. There was a link that was directed to a form where participants could record their water and beverage intake every time they consumed water or beverages. This record was supervised by the research’s team every day; therefore, incomplete or dubious data would be handled immediately. The research’s team reminded participants every day to keep recording their water and beverage intake until the seventh day. Research team was comprised of dietitians who had been trained regarding questionnaire and fluid intake previously.

On the 4th day of the data collection, participants were expected to fill the form of General Physical Activity Questionnaire (GPAQ) for observing participants’ physical activity while pandemic (17). On the last day, the 7th day, participants were required to record 24 h of food consumption. The link of GPAQ and food record was provided on the day before fulfillment.

### Measurements

Data of characteristic, drinking behavior during pandemic, working environment, body weight and height, and hydration knowledge, attitude, and practice were obtained by self-reported questionnaire on the recruitment stage. Characteristic data consisted of sex, age, address, education background, marital status, and ethnic with 10 questions. Alongside, there were also questions regarding medication taken for the last 3 months and diseases that might be present in participants to ensure that they were eligible for this study. For working environment and drinking behavior during pandemic, there were 20 and 22 questions listed for general workers and health workers, respectively. Different set of questions was slightly difference between groups since there was a gap between their working environment. Among health workers, there was questions regarding COVID-19 patient exposure considering they treat patients with COVID-19 that might affect their drinking behavior. As questions for hydration knowledge, attitude, and practice, we listed eight, six, and five questions, respectively. Questionnaire applied during recruitment had been pre-tested before among workers in an unpublished data. The record of name, phone number, and email address were also applied; thus, we could contact the participant for later. Incomplete or unclear data recorded was asked further to the participants after they were contacted for water and beverage intake data collection.

Water and beverage intake was measured using a 7-day fluid record with six questions. The record was conducted online where participants were able to fill the form after they drank every day. Participants filled the data of (1) time, (2) occasion, (3) type of beverage, (4) location of drinking, (5) type of container used, and (6) how many containers consumed. We provided options in the picture for types of container used; therefore, participants could select the container and input the number of containers consumed in data number 6. Later, research’s team calculated the drinking volume by multiplying the volume of the container and the amount of the container used. Questionnaire has been validated and used for hydration studies in Indonesia, in the example in a study by Bardosono et al. (18).

In beverage data, research’s team categorized the beverage into seven types based on Guelinckx et al. that were modified with population adjustment (19). The seven types were water, hot beverages, milk and derivatives, sugar sweetened beverages, juices, alcoholic drinks, and other beverages. Hot beverages included tea and coffee without additional sugar. Whole milk, skimmed milk, yoghurt, and ready to drink milk were included in milk and derivatives, but condensed milk was not because of its high sugar content and low milk content. Sugar sweetened beverages consisted of ready to drink beverages, tea and coffee with additional sugar, soda, isotonic drink, and milk tea or boba drink. Juices were only including 100% juice and alcoholic drinks were including beverages with alcohol content. Other beverages consisted of soy milk, herbal drink, and various sweet Indonesian sorbets case.

On the last day of fluid record, participants were also required to conduct 24-h food record to investigate the nutrient intake. Food record was done through Google Sheet where only individual of participant and enumerator could access. This record was also supervised by enumerators.

The main outcome of this research was daily water and beverage intake while being a health worker was an exposed group. Participants who filled the 7-day fluid record satisfactorily for at least 4 days were included in the study. The daily water intake was categorized based on Indonesian RDA that was released by the Ministry of Health to determine its adequacy (20). Participants were classified into low drinker for consuming less than 1,880 ml/day for women and less than 2,000 ml/day for men. This number was lower by 20% than the actual guidance because 20% of water recommendation was from food that was not recorded in 7-day fluid record.

Body mass index (BMI) was categorized by WHO Western Pacific Region (21). BMI less than 18.5 was categorized into underweight, 18.5–23.0 was grouped into normal, 23.1–25.0 was included into overweight, and 25.1 or more was categorized into obese. Cut off for physical activity referred to GPAQ guideline where metabolic equivalent of task (MET)/week was less 600 categorized into low physical activity. Showing MET/week 600 or more was grouped into fair physical activity.

### Statistical Analyses

The Kolmogorov–Smirnov test was carried out to examine data normality in continuous data. For data that were normally distributed, an independent *t*-test was performed to observe the difference between groups, whereas for not normally distributed data, the Mann–Whitney test was performed. The *p-*value was set to 0.05 to display significance. Median (Quartile 1–Quartile 3) was shown for not normally distributed data. To investigate discrete data, cross-tabulation was carried out to show the demographic between groups. Missing or extreme data were excluded from the analyses.

## Results

There were 246 participants who completed all the data and were analyzed further. The general characteristic was presented in Table 1. A total of health worker participants were 144 (58.5%) and general worker participants were 102 (41.5%). Participants were dominated by women with 76% of the sample. In adherence to Asia Pacific region of WHO, there were 18.7% of overweight participants and 34.1% of obese participants. Between groups, the median BMI shown by general worker and health worker was 22.8 (20.2–26.0) and 23.5 (21.2–27.5), respectively. There was a slight difference between groups, but the gap was not statistically different.

**TABLE 1:** General characteristic of participants.

Characteristic	General workers n (%)	Health workers n (%)	Total n (%)
**Sex**			
Male	75 (73.5)	112 (77.8)	187 (76.0)
Female	27 (26.5)	32 (22.2)	59 (24.0)
Age [Median (Q1 – Q3)]	27 (24 – 31)	28 (24 – 32)	27 (24 – 32)
<27 years old	58 (56.9)	65 (45.1)	123 (50.0)
>27 years old	44 (43.1)	79 (54.9)	123 (50.0)
**Indonesia region**			
Java	92 (90.2)	111 (77.1)	203 (82.5)*
Outside Java	10 (9.8)	33 (22.9)	43 (17.5)
**Marital Status**			
Married	56 (54.9)	79 (54.9)	135 (54.9)
Not married	46 (45.1)	65 (45.1)	111 (45.1)
BMI [Median (Q1 – Q3)]	22.8 (20.2 – 26.0)	23.5 (21.2 – 27.5)	23.2 (20.7 – 26.5)
Underweight	16 (15.7)	8 (5.6)	24 (9.8)*
Normal	36 (35.3)	56 (38.9)	92 (37.4)
Overweight	21 (20.6)	25 (17.4)	46 (18.7)
Obese	29 (28.4)	55 (38.2)	84 (34.1)
Knowledge [Median (Q1 – Q3)]	57.6 (45.5 – 69.7)	66.7 (54.5 – 78.8)	63.6 (51.5 – 75.8)
Low (<median)	69 (67.60)	60 (41.7)	129 (52.4)*
Fair (>median)	33 (32.4)	84 (58.3)	117 (47.6)
MET/Week	600 (90 – 1730)	720 (171 – 2400)	720 (120 – 2160)
Low	52 (51.0)	66 (45.8)	118 (48.0)
Fair	50 (49.0)	78 (54.2)	128 (52.0)
**Working status**			
100% Work from Home (WFH)	18 (17.6)	0 (0.0)	18 (7.3)*
100% Work from Office (WFO)	22 (21.6)	144 (100.0)	166 (67.5)
WFH-WFO	62 (60.8)	0 (0.0)	62 (25.2)
**Total**	**102 (41.5%)**	**144 (58.5%)**	**246 (100.0)**

The p-value = 0.05 was set to indicate significant difference; difference between groups was analyzed using the cross-tabulation test; asterisk sign (*) indicates difference proportion between general workers and health workers.

The amount of daily fluid intake among participants was presented in Table 2. There were 59.8% of general workers who did not drink adequate water. This number was lower among health workers (49.3%). Regarding daily fluid intake, general workers and health workers showed 1,759 and 1,939 ml/day, respectively. This amount was still below the recommendation of the Indonesia Ministry of Health. Water contributed the most to total fluid intake among other types of beverages where the percentage was 84.7%. Sugar sweetened beverage was the second most consumed type of beverage that contributed 10.1% of total fluid intake.

**TABLE 2:** Water and beverage intake between groups.

	General worker n (%)	Health worker n (%)	Total n (%)	*p*-value (OR)
**Daily water intake**				
Inadequate	61 (59.8)	71 (49.3)	132 (53.7)	0.134
Adequate	41 (40.2)	73 (50.7)	114 (46.3)	(1.530)
**Fluid intake [Median (Q1 – Q3)]**				
Total (ml/day)	1759 (1447 – 2396)	1939 (1516 – 2446)	1882 (1473 – 2433)	0.378
Water	1526 (1150 – 2043)	1594 (1155 – 1998)	1556 (1155 – 2014)	0.671
Hot beverages	0 (0 – 67)	0 (0 – 46)	0 (0 – 50)	0.457
Milk and derivatives	0 (0 – 43)	0 (0 – 50)	0 (0 – 45)	0.205
SSB	180 (96 – 337)	180 (90 – 338)	180 (94 – 338)	0.800
Juices	0 (0 – 0)	0 (0 – 0)	0 (0 – 0)	0.295
Alcoholic drinks	0 (0 – 0)	0 (0 – 0)	0 (0 – 0)	0.399
Other beverages	0 (0 – 24)	0 (0 – 3)	0 (0 – 21)	0.430
**Types of water contribution to total fluid intake [Median (Q1 – Q3)]**				
Water (%)	84.3 (74.9 – 91.6)	84.8 (76.0 – 90.9)	84.7 (75.7 – 91.4)	0.642
Hot Beverages (%)	0.0 (0.0 – 2.8)	0.0 (0.0 – 2.3)	0.0 (0.0 – 2.6)	0.383
Milk and derivatives (%)	0.0 (0.0 – 1.9)	0.0 (0.0 – 2.4)	0.0 (2.2)	0.205
SSB (%)	10.9 (4.4 – 17.1)	9.6 (5.3 – 16.6)	10.1 (5.0 – 16.8)	0.897
Juices (%)	0.0 (0.0 – 0.0)	0.0 (0.0 – 0.0)	0.0 (0.0 – 0.0)	0.308
Other beverages (%)	0.0 (0.0 – 1.5)	0.0 (0.0 – 0.6)	0.0 (0.0 – 1.0)	0.343

*SSB is sugar sweetened beverages; water intake was considered inadequate for not achieving Indonesian RDA (1,880 ml/day for woman and 2,000 ml/day for man); p-value = 0.05 was set to indicate significant difference; difference between groups was analyzed using the Mann–Whitney test.*

In Table 3, it was presented that 26.0% of participants worked with level 3 PPE, which was the highest level of PPE application among health workers. Health workers with level 1 PPE were merged with general workers who worked fully from office (WFO) due to their same PPE level that was worn during working hour. Despite its lower level, this group showed the lowest intake of total daily fluid compared to level 2 and level 3 [1,789 (1,443–2,253) ml/day]. This group also presented the highest percentage of whose intake was inadequate (61.2%) compared to other groups. In spite of that, the difference between groups was not significant. Intake of hot beverages and juices between groups was significantly different. Comparing total fluid intake between COVID-19 patient exposure levels within health worker participants, the high risk group showed the highest total fluid intake. This group also presented the lowest percentage of whose intake was inadequate, even though it was not significant. None of the types of beverages was significantly different between groups. Very low risk participants were comprised of general workers who were not at all exposed to patients with COVID-19. Among overall participants, those who fully WFO showed the lowest number whose intake was inadequate although the highest intake of total daily fluid was shown by participants who WFH and WFO partially.

**TABLE 3:** Fluid intake among participants based on PPE level, COVID-19 patient exposure, and working scheme.

Fluid intake based on PPE level (mL/day)				
	**100% WFH and WFH – WFO General worker 81 (32.9%)**	**Level 1 health workers & 100% WFO general worker 49 (19.9%)**	**Level 2 health workers 52 (21.1%)**	**Level 3 health workers 64 (26.0%)**
**Water intake**				
Inadequate	48 (59.3%)	30 (61.2%)	24 (46.2%)	30 (46.9%)
Adequate	33 (40.7%)	19 (38.8%)	28 (53.8%)	34 (53.1%)
Total (mL/day)	1757 (1433 – 2435)	1789 (1443 – 2253)	1973 (1473 – 2556)	1989 (1621 – 2435)*^a^*
Water	1522 (1184 – 2039)	1356 (1091 – 1923)	1643 (1163 – 2013)	1663 (1159 – 2034)*^a^*
Hot beverages	0 (0 – 67)	0 (0 – 25)	0 (0 – 29)	0 (0 – 81)*^b^*
Milk and derivatives	0 (0 – 44)	0 (0- 41)	0 (0 – 71)	0 (0 – 48)*^a^*
SSB	176 (86 – 306)	203 (125 – 391)	178 (82 – 276)	178 (98 – 339)*^a^*
Juices	0 (0 – 0)	0 (0 – 0)	0 (0 – 42)	0 (0 – 0)*^c^*
Alcoholic drinks	0 (0 – 0)	0 (0 – 0)	0 (0 – 0)	0 (0 – 0)*^a^*
Other beverages	0 (0 – 21)	0 (0 – 43)	0 (0 – 26)	0 (0 – 0)*^d^*

**Fluid intake based on COVID-19 patient exposure (mL/day)**				
	**Very low risk (General workers) 102 (41.5%)**	**Health workers with low risk 51 (20.7%)**	**Health workers with medium risk 60 (24.4%)**	**Health workers with high risk 33 (13.4%)**

**Water intake**				
Inadequate	61 (59.8%)	27 (52.9%)	31 (51.7%)	13 (39.4%)
Adequate	41 (40.2%)	24 (47.2%)	29 (48.3%)	20 (60.6%)
Total (mL/day)	1748 (1441 – 2384)	1806 (1524 – 2324)	1969 (1471 – 2503)	2058 (1615 – 2660)*^a^*
Water	1484 (1142 – 2017)	1456 (1148 – 1961)	1594 (1146 – 2052)	1730 (1300 – 2050)*^a^*
Hot beverages	0 (0 – 67)	0 (0 – 46)	0 (0 – 35)	0 (0 – 57)*^a^*
Milk and derivatives	0 (0 – 43)	6 (0 – 55)	0 (0 – 49)	0 (0 – 48)*^a^*
SSB	180 (94 – 325)	193 (96 – 310)	150 (88 – 324)	188 (71 – 378)*^a^*
Juices	0 (0 – 0)	0 (0 – 24)	0 (0 – 0)	0 (0 – 0)*^a^*
Alcoholic drinks	0 (0 – 0)	0 (0 – 0)	0 (0 – 0	0 (0 – 0)*^a^*
Other beverages	0 (0 – 24)	0 (0 – 0)	0 (0 – 24)	0 (0 – 0)*^a^*

**Fluid intake based on working scheme (mL/day)**				
	**100% WFH 18 (7.3%)**	**100% WFO 165 (67.1%)**	**WFH-WFO 63 (25.6%)**	

**Water intake**				
Inadequate	11 (61.1%)	84 (50.9%)	37 (58.7%)	
Adequate	7 (38.9%)	81 (49.1%)	26 (41.3%)	
Total (mL/day)	1620 (1424 – 2498)	1932 (1492 – 2407)	1773 (1420 – 2567)*^a^*	
Water	1484 (1169 – 2005)	1576 (1152 – 1984)	1529 (1175 – 2135)*^a^*	
Hot beverages	0 (0 – 50)	0 (0 – 47)	1 (0 – 67)*^a^*	
Milk and derivatives	0 (0 – 57)	0 (0 – 46)	0 (0 – 43)*^a^*	
SSB	228 (48 – 387)	181 (95 – 341)	87 (164 – 291)*^a^*	
Juices	0 (0 – 0)	0 (0 – 0)	0 (0 – 0)*^a^*	
Alcoholic drinks	0 (0 – 0)	0 (0 – 0)	0 (0 – 0)*^a^*	
Other beverages	0 (0 – 7)	0 (0 – 20)	0 (0 – 21)*^a^*	

*SSB is sugar sweetened beverages; water intake was considered inadequate for not achieving Indonesian RDA (1,880 ml/day for woman and 2,000 ml/day for man); the superscript a indicates no significant difference between groups; the superscript b indicates significant difference between the fourth group with the first and second groups, but not with the third group; the superscript c indicates a significant difference between the third group and other group; the superscript d indicates the significant difference between the first group with other group; the p-value = 0.05 was set to indicate a significant difference; difference between groups was analyzed using the Mann–Whitney test.*

Table 4 shows daily nutrient intake among participants. In macronutrient, general workers presented higher amounts. There was a slight contrast of nutrient intake between groups, but with no significant difference. Except for protein and potassium intake, general workers showed higher intake of other nutrients. In adherence to Indonesia RDA, participants failed to achieve all nutrient sufficiency.

**TABLE 4:** Nutrient intake between groups.

Nutrient	General worker	Health worker	Total	*p*-value
Energy (kkal)	1507 (1086 – 1834)	1299 (967 – 1788)	1363 (1017 – 1803)	0.130
Protein (g)	53 (40 – 73)	54 (38 – 68)	53 (39 – 70)	0.746
Fat (g)	55 (42 – 78)	51 (37 – 78)	54 (39 – 78)	0.159
Carbohydrate (g)	181 (130 – 236)	164 (109 – 218)	167 (115 – 228)	0.080
Sodium (mg)	606 (174 – 1161)	495 (246 – 1126)	524 (216 – 1148)	0.907
Potassium (mg)	1131 (862 – 1625)	1157 (809 – 1729)	1152 (829 – 1670)	0.958
Calcium (mg)	295 (184 – 500)	285 (154 – 533)	285 (172 – 518)	0.812
Magnesium (mg)	194 (120 – 245)	171 (119 – 261)	181 (120 – 257)	0.696
Zinc (mg)	6.3 (4.6 – 8.2)	6.5 (4.2 – 8.2)	6.4 (4.5 – 8.2)	0.978
Fe (mg)	7.2 (4.8 – 9.9)	6.6 (4.1 – 9.7)	6.9 (4.4 – 9.7)	0.390
Vitamin A (mcg)	496.2 (285.9 – 1004.7)	490.1 (255.0 – 895.4)	494.2 (271.4 – 958.6)	0.781
Vitamin B12 (mcg)	2.1 (1.0 – 3.6)	1.5 (0.9 – 3.0)	1.7 (0.9 – 3.3)	0.194
Vitamin C (mg)	20.9 (9.3 – 40.6)	21.2 (10.5 – 46.8)	21.2 (10.0 – 44.2)	0.481
Vitamin D (mcg)	1.0 (0.3 – 3.6)	1.1 (0.2 – 4.2)	1.0 (0.3 – 4.0)	0.893

*The p-value = 0.05 was set to indicate a significant difference; difference between groups was analyzed using the Mann–Whitney test.*

## Discussion

This online survey during the pandemic of over 7 days, water intake was the first one that had been conducted in Indonesia. Workers were one of the groups of population who experienced the environment change that might affect their water intake. Despite the insignificant difference of daily fluid intake between worker groups, this study established that both groups showed inadequate daily water intake [1,882 (147–2,433) ml/day].

This study did not compare with pre-pandemic condition, but Laksmi et al. presented that water intake among adult population in Indonesia was 2,599 (1,827–3,465) ml/day (16). Some recent studies showed varied result of water intake during pandemic. In Turkey, Sahin et al. showed a higher intake of water and beverage among adults, it was 4,925 ml/day. The highest contribution was from water (1,379 ml/day) and followed by hot beverages (507 ml/day) that came after (22). In Surabaya, one of the city in Java Island, 52.7% adolescence displayed adequate water intake (2,000 ml/day) with mean daily intake was 1,972+677 ml/day (23). Al-Domi et al. reported that 31.4% of adult people in Jordan did not achieve their adequate water intake (≤8 cups) during COVID-19 pandemic lockdown (24). Compared with before pandemic, drinking habit showed only minor improvement as in Italy, there were only 18.7% adults displayed positive lifestyle change regarding daily water consumption during lockdown (25). In Lebanon, there was a slight improvement of drinking behavior where 27.2% of participants consumed 8 cups of water or more during pandemic, whereas before pandemic there was only 21.7% (26). Difference of method utilized by other research might be the background why the result of this study was contrast from other studies. Some of other studies adapted the self-administered of questionnaire regarding drinking habit or 24 h food recall, whereas this study used a 7-day fluid record.

During data collection, Indonesia was not under lockdown where people were allowed to go to their activities but with strict health protocols such as wearing masks, avoiding crowds and direct touch, and washing hands regularly. People were allowed to eat at the restaurant and work from the office with 50% occupancy (27). Health workers worked with PPE adjusted with level of COVID-19 patient exposure. Health workers who participated in this study were 100% fully WFO without opportunity of WFH (100% WFO). General workers experienced some diverse working schemes. The first was essential sector workers whose working scheme was similar to health workers (100% WFO), in consequence, this group, constituted the largest percentage (67.5%). The second was non-essential sector that allowed workers to partially WFH and WFO combined ranging from 1 to 4 days in a week (WFO–WFH) that contributed 25.2% of participants. The third was non-essential sector workers who were permitted to 100% WFH that comprised 7.3% of the participant. The non-essential sectors could apply different regulation of working scheme considering the office occupancy was 50% at most.

In comparison between total fluid intake among general workers and health workers, there was no significant difference. Despite the limitations of health workers due to strict PPE during working, they did not show a significantly lower intake of total fluid. The awareness of health workers concerning healthy hydration might be responsible for this practice. As McCotter et al. reported, people who received comprehensive knowledge about the importance of sufficient daily water intake among general practitioners resulted better improvement of hydration practice (28). This study showed there was a significant difference of knowledge level between workers group regarding hydration and health where health workers were more likely to come with better knowledge scores.

Comparing between level of PPE among health workers and general workers who were fully WFO, there was no significant difference of water and beverage intake. Nevertheless, health workers who wore level 3 PPE showed the best drinking behavior compared to other groups. The classification of PPE level was in adherence to the Ministry of Health standard of COVID-19 conduct in Indonesia where it is necessary to wear N95 mask, coverall/gown, boots, goggles, face shield, gloves, head cap, and apron (29). Aswad and Loleh showed that in Indonesia, PPE utilization among nurses affected urine color significantly due to heat and low intake of water during PPE utilization (30). Participants with the highest level of PPE utilization might be aware of their condition where they experienced higher risk of dehydration as Albasheer et al. showed that people who lived in hot climate and high humidity country displayed good dehydration state awareness (31). Consequently, the situation where higher awareness was shown enforce the workers to comply fluid intake recommendation (32). For meeting the adequacy, some health workers displayed a habit of drinking water before and after wearing PPE.

Diverse working scheme among participants showed no significant difference of water and beverage intake. Among three groups, 100% WFH workers consumed the least water and beverage (1,620 ml/day). This group also displayed the highest consumption of sugar sweetened beverage (228 ml/day) compared to 100% WFO group (162 ml/day) and WFO–WFH group (181 ml/day). Study among students in Korea showed different pattern where their consumption of sweetened and soda beverage was lower during the pandemic. The restriction of mobility among Korean was responsible for this change since sweetened and soda consumption associated with eating out (33). That environment was different from this study because the restriction was much reduced and workers had more opportunity to access the restaurant nearby compared to students. Despite of the longer time spent for food and drinking activities shown by people who WFH among adults in United States, they did not specify the type of food and beverage participants frequently consumed (15). The working scheme of 100% WFH might enforce people to drink everything they like and less water because they were less likely to feel thirsty due to air-conditioned room or comfortable circumstances (34). Many people tend to drink when they already to feel thirsty instead of drink before they feel thirsty or drink every 1 to 2 h, despite thirsty is one of the signs of dehydration (35, 36). Moreover, Zeigler reported that during self-quarantine of pandemic, water consumption was often replaced by sweetened beverage (37). Borgatti et al. presented that WFH was significantly associated with eating more food when stressed or boring (38). This might explain why sugar sweetened beverage was more highly consumed among workers who experienced 100% WFH or WFH–WFO working scheme.

Alongside of the water and beverage intake, nutrient intake between general workers and health workers did not display significant difference. In spite of the insignificance, general workers presented the higher intake of calorie and carbohydrate. This result needed to be highlighted considering sugar sweetened beverage contributed to daily total fluid intake as much as 9.6 and 10.9% for health workers and general workers, respectively. Azaïs-Braesco et al. reported that among European adults, added sugar was responsible for 15–21% of energy intake (39). In Spain, 9–75-year-old participants showed that 12% of caloric intake was contributed by beverages where milk, alcoholic, and caloric soft drinks were the beverage types consumed the highest (40). Amid the pandemic, calorie and macronutrient intake among students in Canada was significantly increasing, along with alcoholic beverage decreased (41). In the term of food choices, around 55% of adult participants showed an escalation of processed-food consumption compared to before pandemic (42).

Several limitations presented in this study need to be acknowledged. As the data collection in this study was conducted online, the drinking report of participants might be different from data collection that was conducted directly. The guidance provided by research’s team was carefully reviewed to ensure that it was easy to understand by participants. The research’s team also reminded participants every day to report the drinking activity and guided the participants every time they found difficulties to report their drinking activity. The health workers participating in this study were 100% working from the office, and hence the total participants who were 100% WFH were low. Lastly, this study did not compare the water and beverage intake between before and during pandemic. Participants were not asked about their habits regarding water and beverage intake before the pandemic because of the low validity of participants’ recall ability since the pandemic has been occurring for 1 year. Despite of these limitations, this study can be generalized among Javanese population considering that more than 80% of study sample resided in Java Island as the most populous island in Indonesia.

## Conclusion

In conclusion, this is the first study to analyze the water and beverage intake among general and health workers during COVID-19 pandemic era. It was revealed that during COVID-19 pandemic, workers barely meet the water intake recommendation. This study found no significant difference between health workers and general workers. Nevertheless, health workers showed slightly higher intake of total fluid and water intake and lower intake of sugar sweetened beverages. In comparison based on COVID-19 patient exposure, health workers with the highest risk of COVID-19 patient exposure showed the highest percentage of adequate total fluid intake. The high awareness of health workers exposed to patients with COVID-19 and high risk of dehydration might explain these findings. Further studies are needed to explore drinking behavior after COVID-19 pandemic is diminished in the future to see clearer association between pandemic and water intake.

## Data Availability Statement

The original contributions presented in the study are included in the article/Supplementary Material, further inquiries can be directed to the corresponding author.

## Ethics Statement

The studies involving human participants were reviewed and approved by Prof. Dr. Rita Sita Sitorus, Ph.D., Sp.M(K) from the Ethics Committee of the Faculty of Medicine, University of Indonesia – Cipto Mangunkusumo General Hospital. The Ethics Committee waived the requirement of written informed consent for participation.

## Author Contributions

DS, DC, BM, and DF designed the study and investigated the data collection. DS, DC, BM, NM, WL, and PI analyzed the initial data and manuscript writing. All authors involved in writing process of the manuscript and gave final approval for the submitted versions.

## Conflict of Interest

The authors declare that the research was conducted in the absence of any commercial or financial relationships that could be construed as a potential conflict of interest.

## Publisher’s Note

All claims expressed in this article are solely those of the authors and do not necessarily represent those of their affiliated organizations, or those of the publisher, the editors and the reviewers. Any product that may be evaluated in this article, or claim that may be made by its manufacturer, is not guaranteed or endorsed by the publisher.
