# Supplementary material for: Water and Beverages Intake Among Workers Amid the COVID-19 Pandemic in Indonesia
Source: Front Nutr. 2022 Mar 14;9:832641. doi: 10.3389/fnut.2022.832641 (PMC8967173; doi:10.3389/fnut.2022.832641)
Supplement: Supplementary file 1 [file Image_1.PDF]

Supplementary File 1. Pictures of Containers

Container 1	Container 2
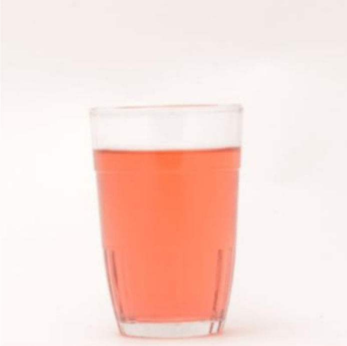	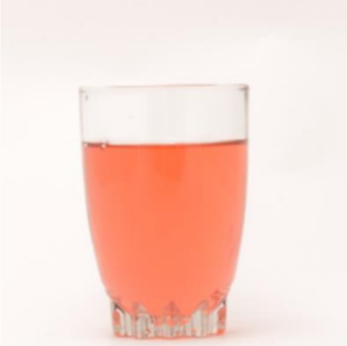
Container 3	Container 4
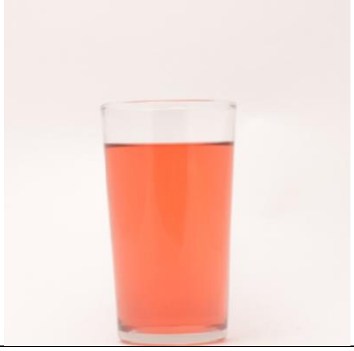	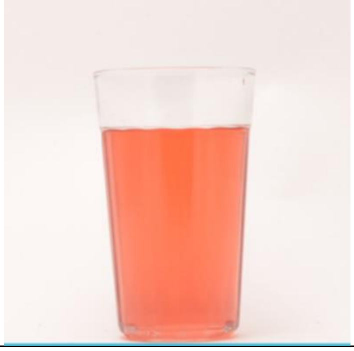
Container 5	Container 6
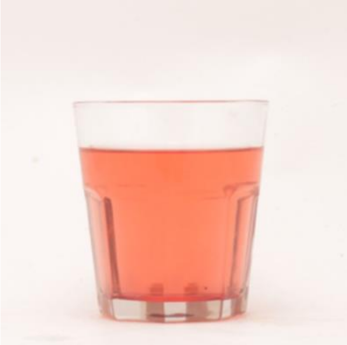	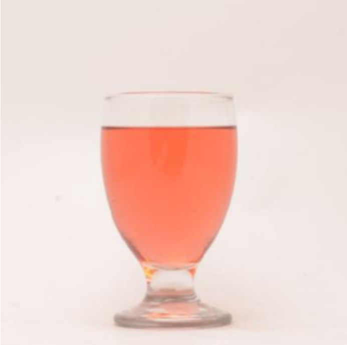
Container 7	Container 8
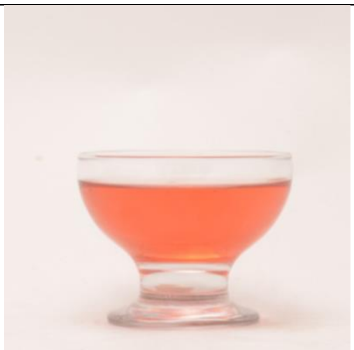	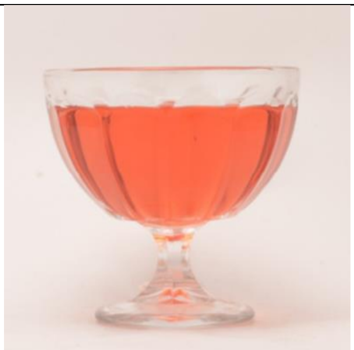

Container 9	Container 10
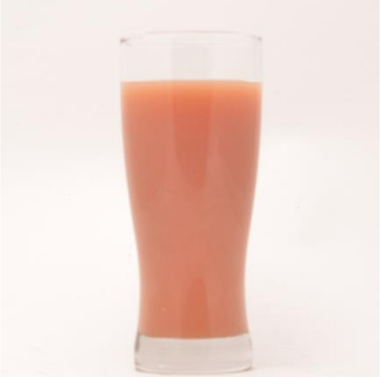	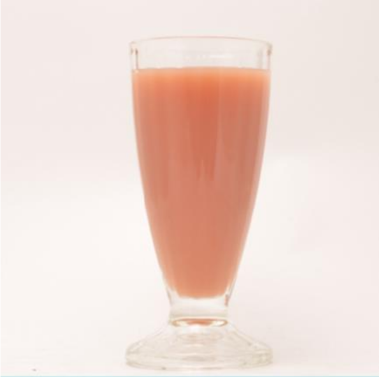
Container 11	Container 12
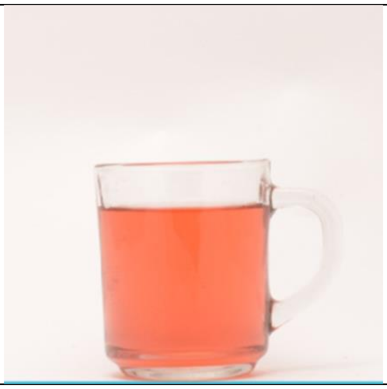	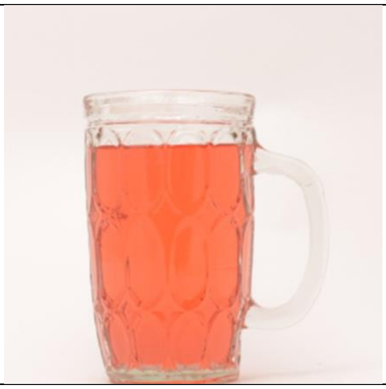
Container 13	Container 14
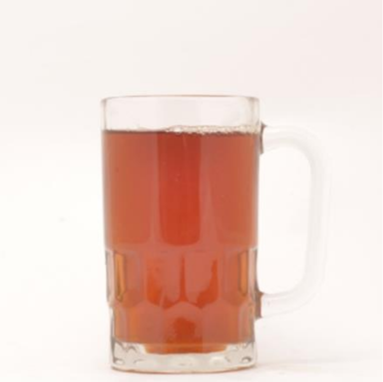	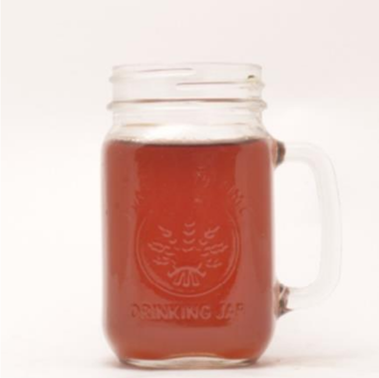
Container 15	Container 16
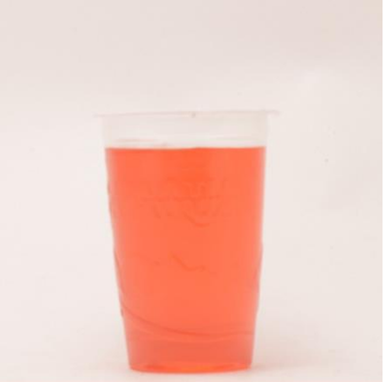	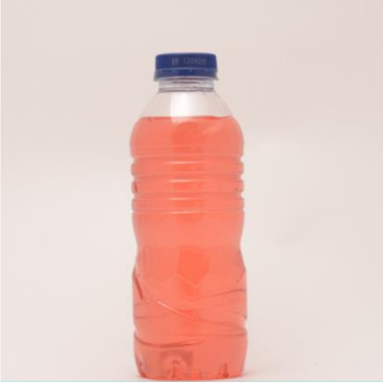

Container 17	Container 18
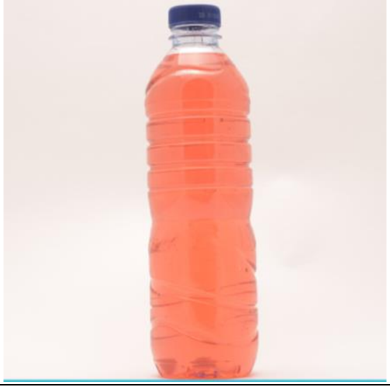	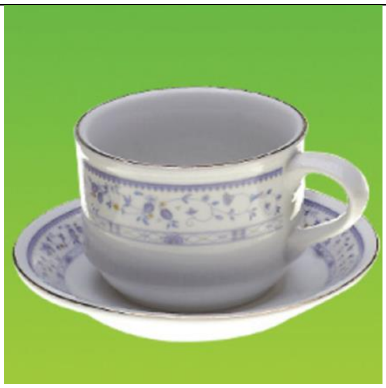
Container 19	Container 20
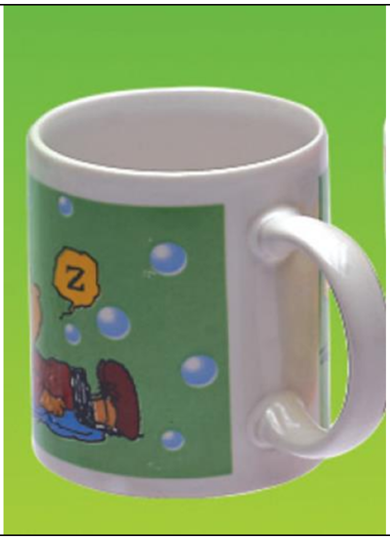	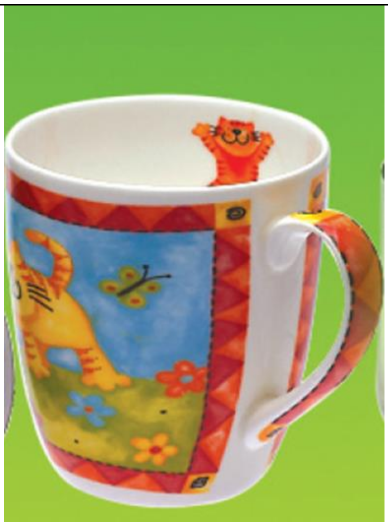
